# Relationship between Younger Age, Autoimmunity, Cardiometabolic Risk, Oxidative Stress, HAART, and Ischemic Stroke in Africans with HIV/AIDS

**DOI:** 10.5402/2011/897908

**Published:** 2011-05-23

**Authors:** Benjamin Longo-Mbenza, Murielle Longokolo Mashi, Michel Lelo Tshikwela, Etienne Mokondjimobe, Thierry Gombet, Bertrand Ellenga-Mbolla, Augustin Nge Okwe, Nelly Kangola Kabangu, Simon Mbungu Fuele

**Affiliations:** ^1^Faculty of Health Sciences, Walter Sisulu University, Mthatha 5117, South Africa; ^2^Department of Internal Medicine, University of Kinshasa, Kinshasa, Democratic Republic of Congo; ^3^Department of Radiology, Kinshasa University, Democratic Republic of Congo; ^4^University of Marien Ngouabi, Brazzaville, Democratic Republic of Congo; ^5^Lomo Medical Cardiovascular Centre for Africa, Limete, Kinshasa, Democratic Republic of Congo

## Abstract

*Background and Purpose*. It now appears clear that both HIV/AIDS and antiretroviral therapy (HAART) use are associated with higher risk of cardiovascular disease such as stroke. In this study, we evaluated the prevalence, the risk factors, and the cardiometabolic comorbidities of stroke in HIV/AIDS Central African patients. *Methods*. This hospital-based cross-sectional study collected clinical, laboratory, and imaging data of black Central African heterosexual, intravenous drug nonuser, and HIV/AIDS patients. *Results*. There were 54 men and 62 women, with a female to male ratio of 1.2 : 1. All were defined by hypercoagulability and oxidative stress. Hemorrhagic stroke was reported in 1 patient, ischemic stroke in 17 patients, and all stroke subtypes in 18 patients (15%). Younger age <45 years (*P* = .003), autoimmunity (*P* < .0001), and metabolic syndrome defined by IDF criteria (*P* < .0001) were associated with ischemic stroke. *Conclusions*. Clustering of several cardiometabolic factors, autoimmunity, oxidative stress, and lifestyle changes may explain accelerated atherosclerosis and high risk of stroke in these young black Africans with HIV/AIDS. Prevention and intervention programs are needed.

## 1. Background

Highly active antiretroviral therapy (HAART) plays a critical role in suppressing viral titers and increasing CD4^+^ lymphocyte counts, which translate to significant reduced morbidity and mortality among HIV patients [1, 2]. It now appears clear that both HIV infection itself and HAART use are associated with higher risk of stroke and metabolic disorders [3–5].

The available information on stroke in African HIV-infected patients comes from South Africa [3]. 

The majority of Central African HIV-infected patients do not have access to drugs of the first line and are not systematically on HAART till now. It is not established whether the HIV infection itself [4], the progression from asymptomatic to symptomatic HIV disease, aging, lifestyle changes, reduction of defining HIV disease infections and tumors, ARV exposure, and traditional and new cardiovascular risk factors directly cause stroke in Central African HIV-infected patients. In these Africans, severe hypertension, hyperuricemia, hyperglycemia, high levels of Dr. dimer and fibrinogen, smoking, excessive alcohol intake, *Helicobacter pylori*, bacterial pneumonia, age ≥ 60 years, pulse pressure ≥ 60 mmHg, low socioeconomic status (SES), and rainy season are the risk factors of stroke [5–8].

The objective of this study was to evaluate the prevalence, the risk factors, and the cardiometabolic comorbidities of ischemic stroke among HIV-infected Central African patients.

## 2. Methods

This cross-sectional study was carried out on HIV-infected patients managed at the Department of Internal Medicine of the Teaching Hospital of Kinshasa, DRC, over a period from 1st January 2004 through 30th May 2008. The Teaching Hospital of Kinshasa is 1500-bed public university hospital that serves a black urban population of 6 million people. The climate is tropical. The study protocol designed according to the Helsinki Declaration II was approved by the local ethics committee. 

### 2.1. Data Collection

Anthropometry recorded weight, height, waist circumference (WC), hip circumference (HC) and body mass index (BMI = weight, kg/height, m²) using standard methods in participants with light clothes and without shoes as described elsewhere [6]. Blood pressure (BP) including systolic blood pressure (SBP), diastolic blood pressure (DBP), and pulse pressure (SBP-DBP) after the participant had rested for 10 minutes seated in a quiet room, was measured in the left arm with elbow flexed at heart level using an Omron HEM 705 electronic BP manometer (Omron Life Science Co. Ltd, Tokyo, Japan). 

Laboratory investigations included haemoglobin, hematocrit, glucose, total cholesterol (TC), triglycerides (TG), HDL-cholesterol (HDL-C), CD4+ count, uric acid, antibodies against oxidized LDL-cholesterol, HIV RNA viral load, serological tests for syphilis, *Toxoplasma gondii*, *Helicobacter pylori (H. pylori)*, herpes simplex virus, *Cytomegalovirus*, *Cryptococcus neoformans*, varicella zoster, bacterial and fungal cultures, D-Dimer, and protein C, cardiac enzymes, protein S, circulating immune complexes, and antiphospholipid syndrome. Glucose levels were measured in fasting plasma samples using glucose-oxidase method and spectrophotometer (Hospitex Diagnostics, Florence, Italy) with internal and external quality control. Total cholesterol, HDL-cholesterol, uric acid, and triglycerides were measured using enzymatic colorimetric methods (Biomérieux, Marcy l'Etoile, France). Antibodies against oxidized LDL-cholesterol were measured using solid phase two-site enzyme immunoassay based on the direct sandwich technique there are two genetic determinants on the oxidized apolipoprotein B molecule (Mercodia AB, Sylveniusgatan 8A, SE754 50, Uppsala, Sweden). *H. pylori* infection was essayed by the determination of IgG antibodies as described elsewhere. CD4+ lymphocyte cell count was performed using CyFlow Counter (Partec GmbH; Munstar, Germany). HIV RNA viral load was recorded using PCR chain reaction (Nuclisens Easy Q HIV-1; Biomérieux, Box tel, The Netherlands). All the Laboratory determinations were documented to be valid and reliable. Cerebrospinal fluid (CSF) was not analyzed for pressure, aspect, and chemistry as lumbar puncture was not indicated in absence of meningitis.

Cardiovascular evaluation comprised clinical symptoms, CKMB, troponin, electrocardiograms (ECG), transthoracic echocardiography, and coronary angiograms. 

Radiological investigations included chest radiograph, brain CT scan, and carotid echo-Doppler studies using 7.5 Mhz transducer of Biosound echographer (Biosound Inc., Indianapolis, Ind, USA) and are described elsewhere.

### 2.2. Definitions

Total obesity was defined by BMI ≥ 25 kg/m² hypertension was defined as SBP ≥ 140 mmHg and/or DBP ≥ 90 mmHg [9] and/or treatment with antihypertensive medication during the previous 2 weeks. Low and high SES were defined according to our previous work [7]. Pulse pressure ≥ 60 mmHg with carotid intima-media thickness ≥1 mm (population median) or carotid plaque was considered a surrogate of arterial stiffness and subclinical atherosclerosis [6]. Diabetes mellitus (DM)/type 2 was defined by either self-report accompanied by use of antihyperglycaemic medication the by elevated serum glucose (fasting ≥126 mg/dL or ≥7.0 mmol/L) or by nonfasting glucose ≥200 mg/dL (≥11.1 mmol/dL) [10]. Acute myocardial infarction/ICD-9 410; ICD-10 121,122 and acute stroke/ICD-9 434; ICD-10 163 or ICD-9 431,432; ICD-10 161,162 were defined with the ICD-9 version and the criteria of ESC/ACC and WHO. Stroke was defined in accordance with WHO criteria as “rapidly developing clinical signs of focal, or at times global “as is coma or subarachnoid haemorrhage; disturbance of cerebral function, lasting more than 24 hours or leading to death with no apparent cause other than vascular origin” [11]. The definitions of ischemic stroke and haemorrhagic stroke were based on the criteria of the National Institute of Neurological Disorders and Stroke. The clinical diagnosis of stroke [12] was confirmed on CT scan. Intracerebral space-occupying lesions other than stroke on CT scan were not considered in the present study. 

As detailed in the IDF report from April 2005 [13], participants with three of the defined criteria were considered as having the metabolic syndrome.

The cardiometabolic consequences included arterial hypertension, type 2 diabetes, myocardial infarction, stroke, long QTc ≥ 0.420 ms, gout/hyperuricaemia, and subclinical atherosclerosis [14–16].

HIV-infection characteristics included ELISA HIV-1 seropositivity, CD4+ count, WHO stages [17], and HIV disease status according to the 1993 CDC classification of HIV disease [18], type, and duration in years of ARV.

### 2.3. Statistical Analysis

Student's *t*-test was performed to assess differences between two means and ANOVA between groups. Either chi-square test with and without trend or Fischer's exact test was used to test the degree of association of categorical variables.

A *P*  value <.05 was considered statistical significant. All statistically analyses of database results were performed with the Statistical Package for the Social Sciences (SPSS for Windows, v.13; Chicago, Ill, USA).

## 3. Results

There were 54 men and 62 women with a female to male ratio 1.2 : 1. The mean age of male patients (45.3 ± 8.5 years; *P* = .132) was similar with that of female patients (42.5 ± 11.2 years). 

Stroke was the first clinical manifestation of HIV infection in 12 patients. Isolated hemiparesis, hemianopia, and aphasia were present in 4 patients, 3 patients, and 10 patients, respectively.

CT scan revealed ischemic stroke in the majority of cases (*n* = 17): 14 cases of infarcts occurred in the anterior circulation and 3 cases of infarcts occurred in the posterior circulation. Only 1 patient had supratentorial hemorrhagic stroke while no hemorrhagic infarcts or venous strokes were reported. Carotid Doppler reports revealed no lesions in the only patient with hemorrhagic stroke and in one patient with ischemic stroke but elevated intima-media thickness in 15 stroke cases. Cerebral angiography was not available.

HIV-related dilated cardiomyopathy, atrial fibrillation, elevated circulating immune complexes, protein C deficiency, protein S deficiency, disseminated intravascular coagulation, D-dimer ≥500 ug/L, elevated highly sensitive C-reactive protein, insulin resistance, elevated level of antibodies against oxidized LDL-cholesterol, and antiphospholipid syndrome were similarly (*P* > .05) frequent in the group with ischemic stroke (*n* = 17) and the group without stroke (*n* = 99).

There was a significant association between lower level of CD4+ count, higher levels of WC, HC, glucose, hematocrit, pulse pressure, and the presence of ischemic stroke (Table 1). 

There was a significant association between high SES males 29.6%,  *n* = 16 versus, 1.6%  *n* = 1 females), physical inactivity, current cigarette smoking, excessive alcohol intake, total obesity, arterial hypertension, congestive heart failure, myocardial infarction, type 2 diabetes, gout, autoimmunity, pulse pressure ≥60 mmHg, first-line ARV exposure, and the presence of ischemic stroke (Table 2). The majority of ischemic stroke cases were at WHO AIDS Stage 4 (*n* = 13/19) and Stage 3 (*n* = 3/12).

## 4. Discussion

We found that the majority (*n* = 17/18) had ischemic stroke and only 1 patient had hemorrhagic stroke in accordance with few reports [3, 19], but contrasting a much higher rate of intracerebral haemorrhage in most of the reported series [20–22]. 

### 4.1. Mechanisms of All Stroke Subtypes

Several clinical, imaging, and autopsy studies have observed a greater-than-chance association between HIV infection and stroke [4, 20–23]. Both types of stroke were frequently associated with concomitant central nervous system disease (opportunistic infections, and tumors) [3]. Intravenous drug abuse infrequently reported as cause of stroke (underlying vasculitis, vasoconstriction, and immunologic mechanisms) from developed countries [19], opportunistic vasculitis, vasculopathy, meningitis, *Toxoplasma gondii, Cytomegalovirus,* syphilis, or *Cryptococcus* [9–15, 19] are not reported in this study. Cardioembolism due to dilated cardiomyopathy, atrial fibrillation, protein S deficiency, protein C deficiency, hypercoagulability, abdominal lymphomas, and disseminated Kaposi's sarcomas were common in this study as reported by other studies [3, 4, 19–23].

### 4.2. Mechanisms of Intracerebral Haemorrhage

Uncontrolled hypertension was identified as the possible cause of hemorrhagic stroke. This situation may be explained by the decline of most of the conditions conferring an increased risk of intracerebral haemorrhage in recent years [19].

### 4.3. Mechanisms of Ischemic Stroke

For the findings to be meaningful, one has to compare our data with those on young black HIV-positive patients [3] and young HIV-negative black African patients [5–8, 24] with stroke. Coagulopathies, insulin resistance, metabolic syndrome, lifestyle changes, and autoimmunity were not reported in young HIV-negative African patients [20, 21]. Coagulopathies and autoimmunity but not insulin resistance and lifestyle changes were reported in young HIV-positive African patients [3]. Our data show that severity of HIV disease, low CD4+ count, insulin resistance, autoimmunity, metabolic syndrome, total obesity, hypertension, type 2 diabetes, gout, myocardial infarction, and ischemic stroke are emerging in Central Africans with HIV/AIDS because of demographic transition, lifestyle changes, low CD4+ count, ARV exposure, and later stages of HIV disease, but not from atherogenic dyslipidaemia [25, 25]. The rates of modifiable risk indicators of ischemic stroke in these HIV/AIDS patients are impressively higher than those frequently reported in uninfected and infected individuals worldwide [26, 27]. The main emphasis in the management of ischemic stroke should be on professionally supervised lifestyle changes, particularly efforts to smoking cessation, reduce body weight and alcohol intake, and increase moderate physical activity. Elevated blood pressure and hyperglycaemia may, however, need additional drug treatment.

Young age, autoimmunity, and metabolic syndrome, identified as independent risk factors of ischemic stroke in this study, may explain atherosclerosis in these black Central African HIV/AIDS patients. Approximately 50% of atherosclerotic coronary artery disease in developed populations occurs in the absence of traditional risk factors, such as smoking, hypertension, diabetes mellitus, and hypercholesterolemia [28]. Over 80–100% of atherosclerotic stroke and coronary heart disease in Central Africans occur with normal lipid profile, hyperuricemia, and infections (*Helicobacter pylori*, and bacterial pneumonia) [5–8]. 

The present study shows that all HIV/AIDS patients had elevated hs-CRP levels and antibodies against oxidized LDL cholesterol in terms of oxidative stress, proinflammatory and chronic inflammation. Chronic inflammation may act independently or synergistically with traditional atherosclerotic risk factors in the pathogenesis of atherosclerotic stroke and may also be associated with a hypercoagulable state (elevated D-Dimer, high hematocrit, protein C deficiency, protein S deficiency) in these HIV/AIDS patients [29, 30]. 

The present findings confirm the unclear role of autoimmunity as directly pathogenic or simple marker of arterial disease in stroke of HIV/AIDS patients. A host tissue component may become antigenic when altered by a drug or proatherosclerotic effects of HIV infection-related endothelial dysfunction [19]. In HIV/AIDS patients, autoantibodies and the associated release of tumor necrosis factor-*α* have been revealed to reduce proteins activity [30]. Elevated antiphospholipid IgG antibody titers and circulating immune complex were found in HIV/AIDS patients with and without ischemic stroke [3, 31]. Insulin resistance is an independent risk factor for atherosclerosis in rheumatoid arthritis [32].

### 4.4. Limitations

The limitations of this study are related to the observational design and cross-sectional nature of the current analyses as well as the small size of stroke cases and the clinical status of the population studied. In this respect, the results reported herein are only associations from which no conclusions regarding causality can be drawn. Further studies with HIV negatives are needed to demonstrate the independent role of HIV infection itself from HAART and metabolic factors. Early assessment of the vascular status, continuous monitoring of drugs, and Preventive Cardiology approach in HIV-infected patients are suggested.

In this hospital-based study, mild or minor ischemic strokes probably went undetected.

## 5. Conclusions

Stroke in patients with HIV/AIDS was not associated with opportunistic infections and tumors. Autoimmunity, younger age, and metabolic syndrome are associated with ischemic stroke. Early assessment of the vascular status, continuous monitoring of drugs, and Preventive Cardiology approach in HIV-infected patients are suggested.

## Figures and Tables

**Table 1:** Association between waist circumference (WC), hip circumference (HC), fasting glucose, hematocrit, pulse pressure, and ischemic stroke among HIV/AIDS patients.

Variables of interest	Presence of ischemic stroke mean ± SD	Absence of ischemic stroke mean ± SD	*P* value
WC (cm)	119.6 ± 5.8	98.7 ± 19.2	<.0001
HC (cm)	118.8 ± 5.1	105.8 ± 16.6	.002
Fasting glucose (mg/dL)	140.4 ± 10.9	116.4 ± 36.9	.010
Hematocrit (%)	39.6 ± 0.04	32.9 ± 7.8	<.001
Pulse pressure (mmHg)	86.1 ± 8	47.1 ± 16.4	<.0001
CD4^+^ count (cells/mm^3^)	107.6 ± 1.7	207.6 ± 179.4	.024
Viral load	132.10^3^ ± 12.10^3^	132.10^3^ ± 258.0^3^	1.000

WC: waist circumference; HC:hip circumference.

**Table 2:** Univariate risk factors of ischemic stroke among HIV/AIDS patients.

Variables of interest	Presence of stroke *n* (%)	Absence of stroke *n* (%)	*P* value
High SES	17 (100)	19 (19.2)	<.0001
Men	16 (94.1)	38 (38.4)	<.0001
Physical inactivity	16 (94.1)	45 (45.5)	<.0001
Smoking	17 (100)	9 (9.1)	<.0001
Excessive alcohol	16 (94.1)	28 (28.6)	<.0001
Total obesity	16 (94.1)	36 (46.2)	<.0001
Hypertension	17 (100)	17 (16.2)	<.0001
Heart failure	16 (94.1)	9 (9.1)	<.0001
Myocardial infarction	16 (94.1)	2 (2)	<.0001
Type 2 diabetes	16 (94.1)	5 (5.1)	<.0001
Gout	16 (94.1)	0 (0)	<.0001
Autoimmunity	16 (94.1)	36 (46.2)	<.0001
Pulse pressure ≥60 mmHg	16 (94.1)	18 (18.2)	<.0001
ARV exposure	17 (100)	34 (34.3)	<.0001
